# Robot-navigated minimally invasive transforaminal lumbar interbody fusion workflow

**DOI:** 10.3171/2025.4.FOCVID2516

**Published:** 2025-07-01

**Authors:** Gianluca Vadalà, Fabrizio Russo, Vincenzo Denaro

**Affiliations:** 1 Operative Unit of Orthopaedic and Trauma Surgery, Campus Bio-Medico University Hospital Foundation, Rome, Italy; and; 2 Departmental Faculty of Medicine and Surgery, Campus Bio-Medico University of Rome, Italy

## Abstract

This video demonstrates the robot-navigated minimally invasive transforaminal lumbar interbody fusion (MIS-TLIF) technique for spondylolisthesis. Navigation and robotics enhance accuracy in pedicle screw placement, reduce operative time and radiation exposure, and improve patient recovery. The case of a 70-year-old patient with L4–5 spondylolisthesis and spinal stenosis is presented. Key surgical steps include intraoperative imaging, preoperative planning, robot-guided screw placement, targeted decompression, and interbody cage implantation. The workflow optimizes safety and efficiency while minimizing tissue disruption. Postoperative imaging confirms precise implant positioning and spondylolisthesis correction, demonstrating how robotics and navigation contribute to superior surgical precision, reproducibility, and improved clinical outcomes.

The video can be found here: https://stream.cadmore.media/r10.3171/2025.4.FOCVID2516

## Transcript

The aim of this video is to describe the surgical technique of robotic and navigated minimally invasive TLIF for the treatment of spondylolisthesis.^1^

### 0:31 Clinical Case.

In this video, we will present the clinical case of a 70-year-old woman affected by low back pain and bilateral leg pain for over 5 years, which was unresponsive to conservative treatments.

### 0:43 Radiographs.

X-rays shows L4–5 spondylolisthesis, which is unstable at dynamic x-ray evaluation.

### 0:52 MRI.

MRI shows spinal stenosis at L4–5 with bilateral facet hypertrophy.

### 1:00 Robotic Arm Setup.

The surgical procedure is aided by a robotic arm, which is solidly fixed to the operative table. The end effector is then attached to the robotic arm.^2^

### 1:12 Neuromonitoring Setup.

The patient is secured in prone position on a carbon fiber floating top radiolucent table and Wilson frame. The neuromonitoring that will aid in the surgical procedure is set up.

### 1:24 Sterile Field Preparation and Instrument Calibration.

The sterile field is then prepped in a way so that the cone beam CT can freely scan the patient. This procedure is done by wrapping the floating table and leaving a free passage underneath. The robotic arm is sterile prepped, and the navigation instruments are calibrated.

### 1:55 Reference Frame Positioning.

Prior to image acquisition, a reference frame is rigidly attached to the PSIS to permit the navigation station to correlate the 3D image to the patient’s position in space.

### 2:07 Intraoperative CT Scan.

The intraoperative cone beam CT scan is brought into position. After 2D AP and lateral x-ray are obtained, the field of view is defined and the CT scan is performed.

### 2:20 Screw Planning.

Following imaging acquisition, preoperative planning is performed. Each level of interest is automatically segmented and labeled. Pedicle screws are automatically positioned by the software. Instrumentation planning can be manually adjusted. Individual screws are positioned along the desired trajectories by visualizing the screw placement in all three dimensions and optimizing the trajectory accordingly in all anatomical planes. Screw length and diameter may also be adjusted.

### 2:53 Robot-Assisted Percutaneous Screw Placement.

The robotic arm is moved into the operative field to guide percutaneous screw positioning. The end effector automatically adjusts according to the preplanned screw trajectory.

### 3:10 Surgical Approach.

Minimal skin and fascia incisions are performed. The trocar is advanced to reach the cortical bone and soft tissues are dilated.

### 3:26 Watch Out for Skiving.

Attention must be paid to avoid skiving.^3^

### 3:34 Robot-Assisted Percutaneous Screw Placement.

The drill guide is then inserted and fixed to the cortical bone locking the system. A depth gauge set on 30 mm of length is used to avoid overdrilling. A drill bit of 2.6 mm is used to drill the pedicle.

### 3:55 Drilling Under Neuromonitoring.

Drilling is performed under neuromonitoring guidance as an additional intraoperative safety check.^4^

### 4:07 K-Wire Insertion.

A K-wire is then inserted deep in the spongious bone, feeling the typical loss of resistance as a tactile feedback, the trocar removed, and the robotic arm moved to the next screw.

### 4:21 Alternative Technique.

The placement of K-wires can also be performed through the so-called "navigated drill guide technique."^5^ The drill guide is aligned with the preplanned trajectory and held in place. The pedicle is then drilled under neuromonitoring. A K-wire is then inserted deep in the spongious bone, feeling the typical loss of resistance as a tactile feedback. After insertion of all the K-wires, they are hold in place to allow decompression, interbody cage implantation, and final screw insertion.

### 5:22 MR and CT Image Fusion.

Intraoperative cone beam CT images performed in prone position are fused with the preoperative MRI performed in supine position. This allows navigation of soft tissue such as disc sequestrum in the spinal canal, ligamentous flavum, helping the surgeon in targeted decompression.

### 5:39 Surgical Decompression.

The navigated probe is used to establish the proper skin incision. A median incision centered on the L4–5 disc space is made, and monoliteral access to the laminae is performed. A minimal detachment and lateral retraction of the paraspinal muscles are performed to expose the posterior elements of the spine. Navigation is used to visualize the correct facet joint to be removed to access the disc space.

### 6:12 Facetectomy.

The superior facet joint is partially removed using a spinal chisel.

### 6:30 Partial Hemilaminectomy.

A partial hemilaminectomy is performed using the high-speed burr first and later with Kerrison rongeurs to create a window access the disc. Inferior facet joint is carefully removed.

### 6:55 Flavectomy.

The ligamentum flavum is removed to expose neurological structures.

### 7:03 Discectomy.

The disc space is then identified. Dural sac, superior and inferior nerve roots are visualized and retracted. The disc space is accessed by a wide disc incision and removal of the annulus. Radical discectomy using disc forceps with different shape and size.

### 7:47 Interbody Cage Sizing.

The height of the disc space is assessed under distraction to decide the proper interbody cage size.

### 7:54 Endplate Preparation.

The intervertebral disc space is thoroughly prepared, ensuring all disc material is removed and the endplates are carefully decorticated to promote fusion.

### 8:08 Cage Trial Assessment.

The trail of the cage is inserted into the disc space to assess the appropriate height, width, depth, and stability to prevent cage migration or subsidence.

### 8:23 Interbody Bone Autograft Placement.

Morselized bone autograft obtained from the lamina and the articular process previously removed is inserted in the interbody space.

### 8:35 Interbody Cage Calibration.

The interbody cage, filled with local morselized bone autograft, is easily calibrated in size and shape using the navigation software.

### 8:48 Interbody Cage Implantation.

The interbody cage is carefully placed into the disc space under careful retraction of superior nerve root cranially and dural sac with inferior nerve root medially. Navigation of the cage allows real-time visualization of the device in the disc space, indicating the exact depth and orientation toward the final position.

### 9:20 Screw Placement.

Following removal of the retractor, pedicle screws are implanted under neuromonitoring using the K-wires.

### 9:38 Postoperative CT Scan.

A final bone beam CT scan is performed to check implant’s placement, listhesis reduction, and segmental lordosis.

### 9:48 Hardware Positioning Compared to Preplanning.

In postoperative 3D CT images, hardware positioning is evaluated and any deviations from the preoperative planning are assessed.^6^

### 10:00 Wound Closure.

The rods are inserted percutaneously through the small distal incision, enlarging it if necessary. Screws are secured with lordotic bars in proper compression, and then wound closure is performed.

### 10:06 Postoperative Radiographs.

Postoperative AP and lateral x-rays show correct implant placement, sagittal alignment, and correction of spondylolisthesis. The patient had a clear improvement in preoperative symptoms, including reduced pain and better neurological function.

## Disclosures

The authors report no conflict of interest concerning the materials or methods used in this study or the findings specified in this publication.

## Author Contributions

Primary surgeon: Vadalà. Assistant surgeon: Russo. Editing and drafting the video and abstract: Russo, Vadalà. Critically revising the work: all authors. Reviewed submitted version of the work: all authors. Approved the final version of the work on behalf of all authors: Russo. Supervision: Russo, Denaro.

## Supplemental Information

### Patient Informed Consent

The necessary patient informed consent was obtained in this study.

## References

[1] Mobbs RJ Phan K Malham G Seex K Rao PJ Lumbar interbody fusion: techniques, indications and comparison of interbody fusion options including PLIF, TLIF, MI-TLIF, OLIF/ATP, LLIF and ALIF J Spine Surg 2015 1 1 2 18 27683674 10.3978/j.issn.2414-469X.2015.10.05 PMC5039869

[2] Vadalà G De Salvatore S Ambrosio L Russo F Papalia R Denaro V Robotic spine surgery and augmented reality systems: a state of the art Neurospine 2020 17 1 88 100 32252158 10.14245/ns.2040060.030 PMC7136092

[3] Basani V Jitpakdee K Härtl R Complications of minimally invasive transforaminal lumbar interbody fusion J Minim Invasive Spine Surg Tech 2023 8 suppl 1 S15 S28

[4] Gautam D Vivekanandan S Mazur MD Robotic spine surgery: systematic review of common error types and best practices Oper Neurosurg (Hagerstown) 2025 28 3 295 302 10.1227/ons.0000000000001293 39037253

[5] Ito M Ueno J Torii Y Utility of a navigated high-speed drill in robotic-assisted screw placement for spine surgery Cureus 2024 16 1 e52779 38389634 10.7759/cureus.52779 PMC10882251

[6] Vadalà G Papalia GF Russo F Intraoperative cone-beam computed tomography navigation versus 2-dimensional fluoroscopy in single-level lumbar spinal fusion: a comparative analysis Neurospine 2024 21 1 76 82 38569632 10.14245/ns.2347106.553 PMC10992660

